# Broadening applications for intrathecal gene therapy: a case for lysosomal storage diseases

**DOI:** 10.1016/j.ebiom.2026.106129

**Published:** 2026-01-14

**Authors:** eBioMedicine

On the Nov 24, 2025, the USA Food and Drug Administration (FDA) approved Itvisma, supported by a pair of clinical trials published in *Nature Medicine* on Dec 8, 2025 (STEER and STRENGTH). Itvisma joins Zolgensma (previously labelled as the world's most expensive medicine) as a gene therapy for patients with spinal muscular atrophy, a progressive neuromuscular disease caused by mutations in a single gene (SMN1). Both Itvisma and Zolgensma comprise an adeno-associated virus serotype 9 (AAV9) capsid containing a corrected copy of the SMN1 gene, but differ primarily in the route of administration.

Zolgensma is injected intravenously, relying on the tropism of AAV9-SMN1 to the CNS and its ability to cross the blood–brain barrier (BBB). However, strong transduction in the liver persists, and severe hepatotoxicity remains a serious concern. Itvisma is injected directly into the cerebrospinal fluid surrounding the spinal cord through the lower back, allowing a more efficient interaction of AAV9 with the target motor neuron cells, and a reduced exposure of off-target tissues.

Notably, Itvisma is the first intrathecal gene therapy ever to receive FDA approval, and it could set a precedent for similar intrathecal AAV9 approaches, targeting a broader range of diseases, to follow in its path. Lysosomal storage diseases (LSDs) are a group of genetic metabolic conditions in which gene therapies are a topic of intense research, yet currently no approaches where the gene therapy is applied *in vivo* have been approved. LSDs are defined by the accumulation of toxic metabolites inside the lysosomes of cells due to dysfunctional proteins arising from inherited mutations, ultimately leading to cell death.

In diseases such as CLN2 disease (neuronal ceroid lipofuscinosis, also known as Batten disease), where progressive neurodegeneration begins as early as age 2 years, replacement soluble purified enzymes can be administered (enzyme replacement therapy [ERT]) directly into the brain. However, this treatment is required to recur every other week for a patient's entire life, and so one-time gene therapy approaches have long been sought. CLN7 disease, similar to CLN2 disease, results in a progressive decline of motor skills, speech, and cognition, typically proving fatal in late childhood. However, the CLN7 protein, being a membrane protein, cannot be functionally replaced through ERT, and thus no disease-modifying treatments currently exist.

Crucial work from Greenberg and colleagues, published in the January, 2026, issue of *eBioMedicine*, reports findings from a phase I clinical trial for a gene therapy against CLN7 disease in four young participants. The trial showed a favourable safety and tolerability profile of intrathecal AAV9-CLN7, despite administering “the highest intrathecal dose utilised in any gene therapy trial to date”, and also represents the first gene therapy approach to be tested in patients with CLN7 disease. So, this early-stage trial, with only the most preliminary hints of efficacy, nevertheless represents an essential step towards a meaningful clinical impact for those with this debilitating disease. A linked commentary highlights further implications of this study in guiding intrathecal dosage limits, immunosuppressive regimens, and therapeutic timing for other diseases of early childhood.

Pompe disease is a LSD where treatment of the once-fatal cardiomyopathy and respiratory failure by intravenous alpha-glucosidase (GAA) ERT has thankfully conferred a longer survival to patients with the infantile-onset form of the disease. However, the inability of ERT to cross the BBB means that progressive CNS manifestations of hearing loss, cognitive delay, speech difficulties, and seizures can emerge, and remain unaddressed by current treatment options.

Completed clinical trials so far have focused on the injection of AAV–GAA therapies locally into the diaphragm or leg muscles. Ongoing trials of intravenously-administered approaches aim for a more systemic effect; however, they remain primarily targeted for the myopathological form of Pompe disease, with interventions designed for muscle (NCT05793307, NCT04174105) or liver transduction (NCT03533673). Yet, to date, very few preclinical studies have explored intrathecal or intracerebroventricular administration of AAV-based therapeutics in mouse models of Pompe disease, with just three published between 2012 and 2018.

Despite promising results, the clinical treatment landscape for young patients with CNS manifestations of Pompe disease remains sparse, and few biomarkers are available to guide the identification of the patients who could benefit most from CNS-targeted gene therapies. A study by Kishnani and colleagues published in the January, 2026, issue of *eBioMedicine* aims to address this, highlighting plasma glial fibrillary acidic protein (GFAP) as closely correlated to CNS disease burden, and defining it as a valuable biomarker in the detection and monitoring of CNS involvement in infantile-onset Pompe disease.

Further, preclinical development of intrathecal gene therapy could be supported by more sophisticated mouse models of infantile-onset Pompe disease, such as that developed by Colella and colleagues, published in *eBioMedicine* in 2020. Unlike earlier models, these mice display early-onset spinal cord defects and respiratory phenotypes more similar to the human disease. As part of the wider field of approaches that circumvent and cross the BBB, and together with the advances made with high-dose intrathecal AAV9 administration, CNS-targeting gene therapies for LSDs are poised for progress.

